# Adjunctive Therapy with Chinese Herbal Medicine Lowers Risk of Hearing Loss in Type 2 Diabetes Patients: Results from a Cohort-Based Case-Control Study

**DOI:** 10.3390/ph17091191

**Published:** 2024-09-10

**Authors:** Hui-Ju Huang, Hanoch Livneh, Chieh-Tsung Yen, Ming-Chi Lu, Wei-Jen Chen, Tzung-Yi Tsai

**Affiliations:** 1 Department of Nursing, Dalin Tzu Chi Hospital, Buddhist Tzu Chi Medical Foundation, Chiayi 62247, Taiwan; 2 Rehabilitation Counseling Program, Portland State University, Portland, OR 97207-0751, USA; 3 Department of Neurology, Dalin Tzu Chi Hospital, Buddhist Tzu Chi Medical Foundation, Chiayi 62247, Taiwan; 4 School of Medicine, Tzu Chi University, Hualien 97004, Taiwan; 5 Division of Allergy, Immunology and Rheumatology, Dalin Tzu Chi Hospital, Buddhist Tzu Chi Medical Foundation, Dalin Township, Chiayi 62247, Taiwan; 6 Department of Chinese Medicine, Dalin Tzuchi Hospital, Buddhist Tzu Chi Medical Foundation, Chiayi 62247, Taiwan; 7 Graduate Institute of Sports Science, National Taiwan Sport University, Taoyuan 333325, Taiwan; 8 School of Post-Baccalaureate Chinese Medicine, Tzu Chi University, Hualien 97004, Taiwan; 9 Center of Sports Medicine, Dalin Tzu Chi Hospital, Buddhist Tzu Chi Medical Foundation, Chiayi 62247, Taiwan; 10 Department of Medical Research, Dalin Tzu Chi Hospital, Buddhist Tzu Chi Medical Foundation, Chiayi 62247, Taiwan; 11 Department of Environmental and Occupational Health, College of Medicine, National Cheng Kung University, Tainan 70428, Taiwan

**Keywords:** type 2 diabetes, hearing loss, Chinese herbal medicine, cohort-based case-control study, risk

## Abstract

Hearing loss is a frequently observed complication of type 2 diabetes (T2D). Emerging evidence has found that Chinese herbal medicine (CHM) can effectively treat chronic disease; nevertheless, it is unclear if adding CHM to the routine management of T2D would modify sequent risk of hearing loss. This cohort-based case-control study was conducted to address this issue. First, a total of 64,418 subjects aged 20–70 years, diagnosed with T2D between 2002 and 2011, were extracted from a nationwide health claims database. Among them, we identified 4516 cases of hearing loss after T2D by the end of 2013. They were then randomly matched to 9032 controls without hearing loss at a 1:2 ratio. Following conditional logistic regression, we found the addition of CHM to conventional care reduced the risk of developing hearing loss, with an adjusted odds ratio of 0.75 (95% confidence interval: 0.70–0.83). Specifically, taking CHM products for at least two years benefits T2D patients in lowering sequent risk of hearing loss. The findings herein implicated that integrating CHM into conventional care substantially correlated to lower risk of hearing loss for T2D patients, but further basic research is needed to secure the application of finished herbal products.

## 1. Introduction

Today, chronic illness has been regarded as a leading health-related challenge and is a major cause of both death and disability worldwide. For example, there are nearly 530 million people living with diabetes in 2021 worldwide, and approximately 96% of those cases are type 2 diabetes (T2D) [1]. These growing cases of diabetes place a substantial burden on health-care systems. One recent estimate by the International Diabetes Federation (IDF) shows that present-day health expenditures for the care of diabetes was US$966 billion globally and is forecasted to reach more than US $1054 billion by 2045 [2]. On average, the per-capita healthcare costs for people with diagnosed with T2D is $19,736 US, nearly triple that of those without this condition [3]. 

Apart from the enormous economic burdens mentioned above, the intrinsic metabolic changes accompanying T2D might be responsible for a wide array of comorbid conditions commonly seen in T2D, including hearing loss. One recent study by Shafiepour and colleagues found that about two-thirds of adults with T2D experienced auditory dysfunction in ancient treatments [4]. Other research has found that people diagnosed with diabetes were more than twice as likely to suffer from hearing impairment as those without diabetes [5]. Hyperglycemia, which is the central cause of diabetes, may insidiously create chemical changes throughout the body that leads to neuronal damage. Reactive oxygen species (ROS) induced by hyperglycemia would lead to the activation of nuclear factor kappa beta (NF-κB) signaling by amplifying the function of caspase family [6,7]. At present, converging scientific consensus is that NF-κB activity is central to the inflammatory response. These include tumor necrosis factor-α (TNF-α), interleukin-1β (IL-1β), and IL-6 [8], all of which play a critical role in the pathogenesis of central auditory dysfunctions [9,10]. For diabetic patients, the concurrent hearing loss can impair speech communication and socialization and thus affect their mental health and quality of life. Notably, prior evidence revealed a positive correlation between hearing loss and suicidal thoughts [11]. With this in mind, adding complementary treatments or interventions to conventional treatment to prevent hearing loss driven by innate inflammatory milieu from hyperglycemia should be explored.

Currently, Chinese herbal medicine (CHM), a special therapy within traditional Chinese medicine, is widely employed in treating or preventing human disease. Several experimental studies have explored the underlying biological activities regulating inflammatory and oxidative stress. For example, one earlier model showed that usage of Bei-Mu correlated with the downregulation of nitric oxide (NO) and pro-inflammatory cytokines [12]. Some multi-herb formulae, like Ping-Wei-San and Ban-Xia-Xie-Xin-Tang, have been indicated to possess beneficial pharmacological activities that included immunostimulant and anti-inflammatory effects, and hence may have the potential as an adjuvant in the treatments for metabolic disorders [13,14]. Despite the reported progress, we still have failed to come up with an alternative therapeutic strategy for preventing or delaying the emergence of auditory dysfunction for those living with T2D. To bridge the gap in existing therapy, we embarked on this cohort-based, case-control study to determine if adding CHM to conventional treatment could reduce the risk of hearing loss in patients with T2D.

## 2. Results

**Figure 1:**
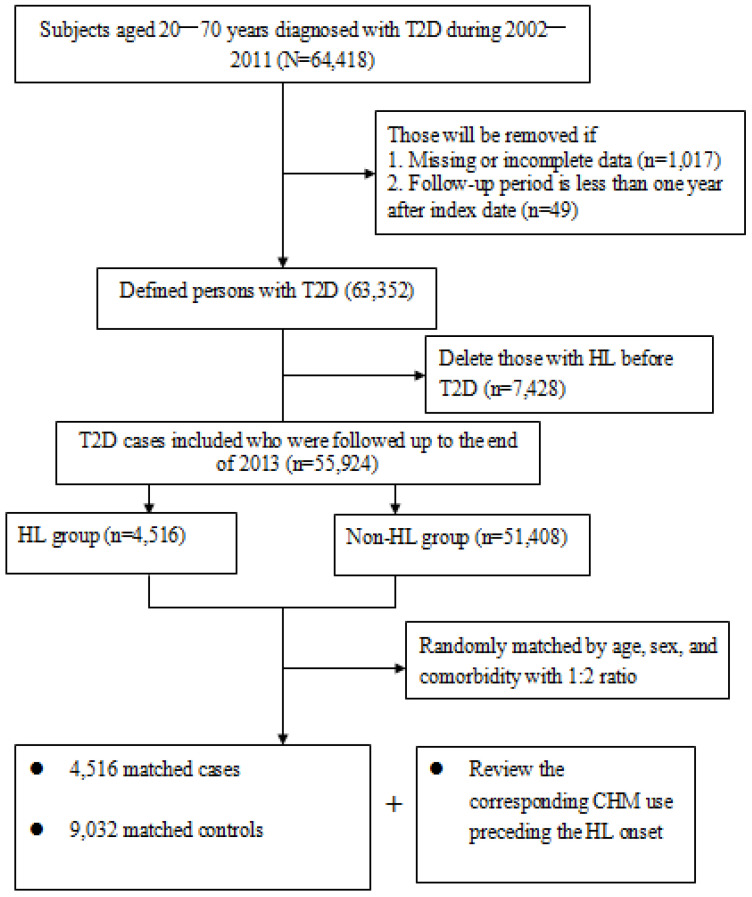
Flowchart of subject selection.

A total of 13,548 subjects (4516 new cases of hearing cases and 9032 randomly matched controls) were included in the final data analysis (Figure 1). The mean age for both groups was 57.6 years and the mean follow-up period was 7.9 years. Throughout the study period, 29.5% of the cases and 35.2% of the controls ever received CHM treatments. On the whole, over half of enrollees were at the middle-income level and lived in cities. No significant baseline differences existed with regards to demographic data or comorbidities between the two groups. Details of the relevant variables at baseline are shown in Table 1.

Using conditional logistic regression, we observed that those who had a history of CHM use in addition to conventional treatment indeed exhibited a reduced risk of hearing loss in comparison to those who had never used CHM (adjusted OR = 0.75; 95% CI: 0.70–0.83). We also noted that the longer the duration of CHM treatment, the lower the hearing loss risk (Table 2). For example, patients using CHM for at least two years experienced a 42% risk reduction after adjusting for demographics and other clinical characteristics at baseline, implying an exposure–response inverse relationship between CHM use and hearing loss. Collectively, such therapeutic benefits of CHM were present regardless of age or sex (Table 3).

**Figure 2:**
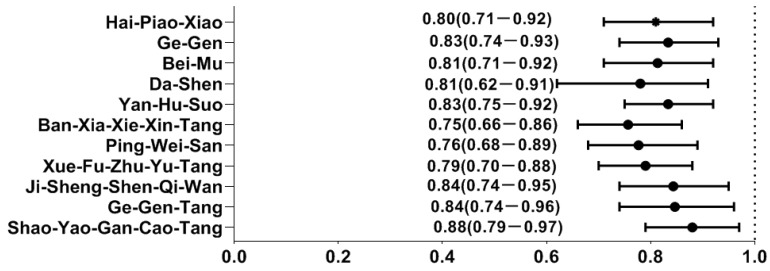
Hearing loss risk determined by multivariate conditional logistic regression across different herbal products. *Y*-axis: Chinese herbal medicines; *X*-axis: odds ratio.

By utilizing the forest plot, we summarized the top ten most commonly prescribed single-herb and multi-herb formulae for the treatment of T2D, along with their associated OR against the risk of hearing loss. Out of these, a total of eleven CHM remedies were correlated to a lower likelihood of hearing loss: Bei-Mu, Ge-Gen, Yan-Hu-Suo, Dan-Shen, Hai-Piao-Xiao, Shao-Yao-Gan-Cao-Tang (SYGCT), Ban-Xia-Xie-Xin-Tang (BXXXT), Ping-Wei-San (PWS), Ji-Shen-Shen-Qi-Wan (JSSQW), Ge-Gen-Tang (GGT), and Xue-Fu-Zhu-Yu-Tang (XFZYT) (Figure 2). The relevant ingredients of herbs reported herein is elucidated on Table 4. 

## 3. Discussion

Chronic hyperglycemia can cause auditory neuropathy associated with damaged blood vessels and nerves in the inner ear. Today, accumulating evidence shows that people with T2D have a higher risk of hearing loss compared to healthy controls [4,5]. Considering that there are currently no effective therapies for this condition, it is of great importance to understand the impact of CHM on the prevention of incident hearing loss in people with T2D. This cohort-based, case-control study found that T2D patients who receive CHM, combined with conventional care, had a 25% lower chance of developing hearing loss. Notably, a longer duration of CHM treatment was correlated with a remarkable decease in the risk of hearing loss. Despite a lack of comparable studies, the current results should enrich and expand prior literature that supports the benefit of CHM in treating chronic disease [15,16].

After stratification according to age and sex, we observed that females benefited more from CHM treatment than males. There may be several reasons why CHM treatment appeared to be more beneficial in females than in males for the prevention of hearing loss. First, an expanding body of evidence on gender differences concludes that females are more interested in health-related information and more likely to engage in healthy behaviors than males [17,18]. In this case, females may be more willing to follow the prescribed medication regimen as directed by their healthcare provider which, in turn, lowers the subsequent risk of adverse events. Additionally, the release of sex hormones, particularly estrogen, may partially explain this beneficial phenomenon. The female hormone estrogen has been shown to protect the inner ear by amplifying the expression of insulin-like growth factor 1 in the cochlea via the phosphatidylinositol 3′-kinase(*PI3K*)-*Akt* signaling pathway [19], which plays a crucial role in the survival and proliferation of neuronal cells [20]. 

Another focal contribution of this study was to identify the specific CHM constituents likely to be related to a lower risk of hearing risk in T2D patients. Of the commonly used single-herb products to treat T2D, the most beneficial effects were seen with Bei-Mu, Ge-Gen and Yan-Hu-Suo. The mechanisms with which these herbal products protected against the onset of hearing loss may primarily pertain to the modulation of inflammatory mediators. Based on former reports from the lipopolysaccharide (LPS)- stimulated RAW 264.7 cell model, these herbs were found to inhibit nitric oxide (NO) and regulate the production of proinflammatory cytokines, such as IL-1β, IL-6, and TNF-α, via the suppression of NF-κB activation [21,22], all of which are involved in the pathogenesis of central auditory dysfunction [9,10]. 

Additionally, both uses of Dan-Shen and Hai-Piao-Xiao were efficient in getting rid of the chance of hearing loss. Dan-Sen is a widely-used single-herb formulation for cardiovascular disease. Tanshinone, a major compound extracted from Dan-Shen, was discovered to possess antioxidant, anti-tumor, and immune-boosting properties by regulating NF-κB and the MAPK pathway in Neuro-2a cells [23]. Hai-Piao-Xiao, on the other hand, has been found to possess neuroprotective properties [24]. In a randomized control trial, a major ingredient of this herb, chitosan, contributed to peripheral sensory nerve regeneration through its profound antioxidant properties [25]. A late meta-analysis focusing on chitosan-injected mice models showed that chitosan exerted a substantial anti-inflammatory effect on active molecules, like TNF-α and IL-1β, via the NF-κB pathway [26]. The pharmacological effects derived from these single-herb formulae collectively might partially explain their beneficial effects on hearing loss in T2D patients. 

Among the multi-herb products commonly used to treat T2D, we identified some herb formulae associated with a decreased risk of hearing. SYGCT, for example, has been shown to improve the decreased phosphorylation of glycogen synthase kinase (GSK)-3β among patients with chronic diseases [27]. Today, GSK-3β has been shown to play a role in the regulation of multiple cellular functions, like metabolism, cell motility, apoptosis, and proliferation of inner ear cells [28]. Recent evident found that the GSK-3β inhibitors CHIR99021 and bFGF had the greatest effect on the release of Lgr5-GFP progenitor, which plays a crucial role in the improvement of cochlear hair cell regeneration [29]. In addition, the reason that BXXXT and PWS were commonly prescribed among subjects included in this study is because diabetic patients often suffer from mild to severe gastrointestinal symptoms [30]. Notably, the pharmacological effects of BXXXT, including anti-emetic, anti-diarrheal, anti-oxidative, anti-inflammatory, and anti-lipidemic effects, have been illustrated in modern pharmacological studies [31], thereby lessening the risk of hearing loss at the same time. Furthermore, one former study using an animal experimental model found that the extract of Magnolia officinalis, a major ingredient purified from PWS, could regulate the production of inflammatory mediators through immunoregulatory phagocytosis via the MAPK signaling pathway [32,33]. All of these may point to the possible mechanisms for these herbs.

We also identified several herb products that may have potential for preventing hearing loss, like JSSQW, XFZYT, and GGT. All of these remedies have been shown to regulate cellular responses to inflammation, which in turn decrease the risk of auditory dysfunction after diabetes onset. Regarding JSSQW, it has been demonstrated to be beneficial in ameliorating insulin resistance via the NO pathway [34]. It is well known that an increase in the formation of free radicals, specifically NO, may be linked to necrotic cell death throughout the body [20]. Regarding the positive impact of XFZYT, as compared to untreated controls, rats fed with this herb were found to have notably reduced levels of inflammatory milieu through the inhibition of the PI3K-AKT-mTOR pathway [35]. Beyond that, this inflammasome signaling has been recognized as an important pathway in regulating autophagy and apoptosis in the inner ear cochlea [20]. Lastly, we found positive effects for GGT in preventing hearing loss among T2D patients. A recent review article outlined that puerarin, a major bioactive ingredient isolated from GGT, can significantly inhibit the expression of pro-inflammatory cytokines through the down-regulation of NF-κB/p65 activation and the inhibition of NF-κB activity [36]. The anti-inflammatory effects of these polyherbal formulations may explain the reduction in the development of hearing loss among subjects with T2D. 

Despite being a pioneering work, some noteworthy limitations remain. First and foremost, this study was a retrospective analysis based on ICD-9-CM diagnosis codes. *M*isclassification *error may result in* biased estimates of model parameters. Nevertheless, NHI periodically reviews random charts to verify the accuracy of claims and medical charge data. We believe that misclassification of cases is always present in administrative database research, but this is expected to occur randomly, which in turn gives a conservative estimate of the overall proportion of null *p*-values. Second, as all data in this study were extracted from a claims-based database, several key factors, such as lifestyle behaviors, family history, and biochemical data, were not available for this study. Thus, residual confounding factors may partly bias the results. Third, information on T2D severity was not available from the database, as we cannot retrieve the relevant laboratory tests from the database. With this aim, we embarked on a sensitivity analysis in which we excluded T2D patients with some comorbidities at baseline, particularly the procedural codes for blindness, amputation, severe kidney failure, hyperglycemia, and hypoglycemia. Upon reanalysis, we still found a beneficial effect of CHM added to existing treatment against the onset of hearing loss, with an adjusted OR of 0.79 (95% CI: 0.72–0.89). Therefore, a bias introduced through diabetic severity is not likely to affect our conclusions. Notwithstanding these limitations, this work is reflective of a population-based investigation that evaluates the association of CHM use with hearing risk in T2D persons via a nationwide health claims database, thus leaving little room for non-response or loss to follow-up. Another strength of this work is the long observation period. With auditory disorder being a major contributor to disability and reduced quality of life, the claims database has the advantages of a 10-year observation period and a large sample size, both of which contributed to a better understanding of the links between diabetes, hearing loss and the effects of CHM. Lastly, the cohort study with a nested case-control approach is an excellent alternative to cohort analysis for determining the causal effects of treatment involved, especially when both treatment and confounding variables may vary over time and when time-varying confounding variables can be influenced by prior treatment or exposure. Hence, the merits of this study may be comparable to the pharmacoepidemiology approach that was carried out under controlled conditions with random allocation of interventions to the groups. 

## 4. Materials and Methods 

### 4.1. Data Source 

All population-level data used in this cohort-based, case-control study was from the Longitudinal Health Insurance Database, which covers one million members randomly selected from National Health Insurance (NHI) in Taiwan [37]. With random extraction from all beneficiaries under the NHI program stratified by age and sex, this database was capable of providing a representative sample of the Taiwanese population. The database included encrypted patient data that documented sex, date of birth, medical diagnoses shown with the International Classification of Diseases, Ninth Revision, Clinical Modification (ICD-9-CM) codes, dates of hospital admission and discharge, medical procedures performed, and prescribed medications, including the use of Chinese herbal products. As the data used have been anonymized prior to being released to each researcher, the individual identifying information at any level cannot be obtained from the database. This work was approved by the facility’s institutional review board (No. B10004021-3).

### 4.2. Underlying Cohort Establishment 

For this study, we identified 64,418 subjects aged 20 to 70 years from the database and primarily focused on those who had ever sought relevant healthcare services due to T2D between 2002 and 2011 (ICD-9-CM code of 714.0). Patients were classified as T2D and included in the analysis if they had at least one admission code for diabetes or three or more outpatient codes within a 365-day period. The date of the first diagnosis of T2D was viewed as the cohort entry date. To adhere to established research procedures, patients with hearing loss before cohort entry, having been followed for less than one year, or having incomplete data were excluded (n = 8494). The remaining cases were followed up until the earliest occurrence of either hearing loss, death, or by 31 December 2013.

### 4.3. Ascertainment of Case and Control Groups 

The primary outcome was the diagnosis of hearing loss that occurred after T2D onset between 2003 and 2013. Participants were defined as having hearing loss if they had ever received at least two outpatient clinic visits in one year or one hospitalization due to this condition during the study timeframe, as reflected in the use of ICD-9-CM codes 388.2, 388.4, 389.2, 389.9, 389.00, 389.10, or 389.12. The first medical visit of hearing loss was viewed as the index date. Afterwards, each case was randomly matched to two controls without hearing loss on the basis of age, sex, and comorbidities (Figure 1). An index date was assigned to each of these controls corresponding to the date of hearing loss diagnosis in the study group, thus ensuring the identical observational probability in all recruited subjects during the study period.

### 4.4. Identification of CHM Exposure 

Recognition of CHM treatment exposure was based on personal medical records for Chinese medicine practitioners that occurred from the cohort entry to the preceding index date. In this study, CHM users were identified if they had ever received relevant CHM treatments for T2D or its relevant symptoms for more than 30 days. To meticulously assess the exposure–response impact of CHM in the prevention of hearing loss, CHM users were further split into three subgroups based on their prescription days within the study period. 

### 4.5. Measurement of Covariates 

Covariates in the statistical model included sex, age, prior medical comorbidities, individual monthly salary, and urbanization of residential area. We first estimated the premium payment category as a proxy of monthly salary, then transformed that to the 25th, 50th, and 75th percentile. In addition, one widely adopted urban–rural classification was used to classify the urbanization of enrollees’ residence [38]. This indicator was based on several dimensions of urbanity, such as population density per square kilometer, proportion of persons with college or above educational levels, percentage of the population over 65 years old, proportion of employment in agriculture, and number of medical doctors per 100,000 inhabitants. Then, three groups were designated as urban, suburban, and rural. Medical comorbidities were defined as a condition leading to at least one inpatient visit or two outpatient visits within one year preceding the cohort entry date. The Charlson–Deyo comorbidity index, an approach that took into account both the number and severity of 17 pre-defined comorbid conditions, was used to quantify participants’ disease burden from comorbidities [39]. 

### 4.6. Evaluation of Data 

This study utilized SAS software for Windows Version 9.3 to perform the statistical analysis. Descriptive statistics, such as mean, standard deviation [1], frequency, and percentages, were applied as appropriate. Comparisons of demographic and clinical data between groups with or without CHM use were carried out using the student’s *t*-test test for continuous variables or chi-square test for categorical variables, as appropriate. Afterwards, conditional logistic regression was used to calculate odds ratios [OR] with 95% confidence intervals (CI) for the association between CHM usage and subsequent hearing loss incidence. In this process, both crude OR based on a simple model with only use of CHM and adjusted OR for a full model with all the variables shown in Table 1 were presented to better understand how the adjustments affect the impact of CHM treatment. In the meantime, we attempted to outline the 10 most commonly prescribed formulae among the enrolled T2D patients. In all statistical tests, a *p*-value of 0.05 or lower was considered significant.

## 5. Conclusions

This is the first work to exploit the cohort-based case-control study to assess the impact of CHM use on the prevention of hearing loss amongst T2D patients. We found that integrating CHM into conventional treatment for diabetes would get rid of the subsequent risk of developing hearing loss by 25%. Furthermore, this therapeutic effect appears to be dose-dependent, with more intensive CHM treatment further reducing the risk for hearing loss. Based on the belief “prevention in itself is preferable to cure”, our study suggests that the integration of CHM into routine care of underactive diabetes mellitus may allow for early prevention of hearing loss. At the same time, these encouraging findings provide an impetus for further research that focuses on the adoption of CHM products, as well as in vivo studies, to explore the beneficial mechanisms of CHM in the treatment of chronic, life-changing diseases. Given the high and rising prevalence of hearing loss among T2D groups, for healthcare providers the urgent need to routinely screen for hearing dysfunction and institute complementary therapy for diabetic disease management should be highlighted.

## Figures and Tables

**Table 1:** Demographic data and selected comorbidities of the two study groups.

Variables	Number (%)	Cases	Controls	*p*
		n = 4516 (%)	n = 9032 (%)	
Age (years)				0.79
≤50	3052	1011 (22.4)	2041 (22.6)	
>50	10,496	3505 (77.6)	6991 (77.4)	
Mean (SD)	57.5	57.6 (9.0)	57.4 (7.3)	0.61
Sex				0.99
Female	6651 (49.1)	2217 (49.1)	4434 (49.1)	
Male	6897 (50.9)	2299 (50.9)	4598 (50.9)	
Monthly income				0.89
Low	5001 (36.9)	1655 (36.6)	3346 (37.0)	
Median	7710 (56.9)	2579 (57.1)	5131 (56.8)	
High	837 (6.2)	282 (6.2)	555 (6.1)	
Residential area				0.21
Urban	7834 (57.8)	2622 (58.1)	5212 (57.7)	
Suburban	2028 (15.0)	643 (14.2)	1385 (15.3)	
Rural	3686 (27.2)	1251 (27.7)	2435 (27.0)	
CCI	6.4 (8.1)	6.5 (8.1)	6.3 (8.2)	0.33

SD: standard deviation, CCI: Charlson–Deyo comorbidity index.

**Table 2:** The association between hearing loss and use of CHM treatment among T2D patients.

CHM Exposure	Patients				Crude OR (95% CI)	Adjusted OR * (95% CI)
	Cases n = 4516		Controls n = 9032			
Non-CHM Users	3184	70.5	5851	64.8	1	1
CHM users	1332	29.5	3181	35.2	0.76 (0.71–0.83)	0.75 (0.70–0.83)
Group 1 (31 days–1 year)	1138	25.2	2622	29.0	0.80 (0.74–0.87)	0.79 (0.73–0.86)
Group 2 (1–2 years)	123	2.7	348	3.9	0.65 (0.53–0.80)	0.64 (0.52–0.80)
Group 3 (more than 2 years)	71	1.6	211	2.3	0.60 (0.47–0.81)	0.58 (0.47–0.79)

* Adjusted for potential confounders including age, residential area, monthly income, and CCI. CHM: Chinese herbal medicine; OR: odds ratio.

**Table 3:** Risk of hearing loss among T2D patients with and without exposure to CHM use, stratified by age and sex.

Variables	Patients, n (%)	Crude OR (95% CI)	Adjusted OR (95% CI)
Female			
Non-CHM users	1515 (68.3)	1	1
CHM users	702 (31.7)	0.68 (0.62–0.76)	0.66 (0.60–0.74) *
Male			
Non-CHM users	1669 (72.6)	1	1
CHM users	630 (27.4)	0.86 (0.77–0.96)	0.86 (0.76–0.95) *
Aged ≤ 50 years			
Non-CHM users	685 (67.8)	1	1
CHM users	326 (32.2)	0.70 (0.60–0.83)	0.69 (0.58–0.81) ^†^
Aged > 50 years			
Non-CHM users	2499 (71.3)	1	1
CHM users	1006 (28.7)	0.78 (0.71–0.85)	0.78 (0.72–0.84) ^†^

* Adjusted for age, residential area, monthly income, and CCI. ^†^ Adjusted for sex, residential area, monthly income, and CCI. CHM: Chinese herbal medicine; OR: odds ratio; CI: confidence interval.

**Table 4:** The ingredient herbs contained in the most-used single-herb and multi-herb products among T2D persons.

Chinese Herbal Product	Ingredients or Generic Name	Functional Classification
Single-herb products		
Yan-Hu-Suo	*Corydalis yanhusuo*	Used to treat Qi stagnation, blood stasis, chest pain, abdominal pain, amenorrhea, dysmenorrhea, and postpartum stasis
Da-Shen	*Salvia bowleyana*	Invigorates the blood and dispels stasis: for a wide variety of blood stasis disorders in any part of the body. Most commonly used for problems affecting the lower abdomen, chest, or hypochondria.
Tian-Hua-Fen	*Radix Trichosanthis*	Its functions are to clear lung heat, dissolve phlegm, relieve toxicity, and expel pus.
Bei-Mu	*Fritillariae Thunbergii Bulbus*	Eliminate phlegm by cooling, moisten lung to arrest cough, and remove stasis to reduce swelling
Ge-Gen	*Kudzu Root*	Acts to expel pathogenic factors in the muscles to clear heat, vent rashes and nourish the fluids to stop thirst. It also works to raise Yang-Qi and alleviate diarrhoea.
Huang-Qin	*Scutellaria baicalensis*	As an adjuvant therapy of inflammation, diabetes, hypertension, different kinds of cancer and virus related diseases.
Mai-Dong	*Ophiopogon Root*	Nourish yin deficiency, promote body fluid production, nourish the lungs, relieve the mind, and eliminate heart fire.
Shyuan-Shen	*Scrophularia ningpoensis*	Clearing away heat and cooling blood, nourishing Yin and reducing fire, detoxicating and resolving a mass.
Jie-Geng	*Radix Platycodi*	It supports respiratory health and benefits the throat. Jie Geng contains anti-inflammatory, anti-bacterial, expectorant and immune boosting properties.
Hai-Piao-Xiao	*Endoconcha Sepiae Os Sepiae seu Sepiellae*	Controlling acidity, harmonizing the stomach, and alleviating pain
Chuanniuxi	*Cyathula Root*	Activates blood, eliminates stasis. Strengthens tendons and bones. Promotes urination, treats dysuria.
Multi-herb products		
Shu-Jing-Huo-Xie-Tang	*Tang-kuei root, White peony root, Corydalis root, Chin-chiu, Cnidium root, Raw rehmannia root, Peach kernel, Hoelen fungus, Atractylodes root, Citrus peel, Notopterygium root, Fragrant angelica, Scabrous gentiana root, Fang feng root, Achyranthes root, Ginger root, Chinese licorice root*	Clears Heat, cools the blood, nourishes Yin and generates fluids. Breaks up blood stasis and invigorates blood circulation. Strongly dries dampness, tonifies the spleen, induces sweating and expels wind-aampness. Promotes urination and leaches out dampness.
Jia-Wei-Xiao-Yao-San	*Bupleurum Root, Chinese Angelica Root, White Peony Root, White Atractylodes Rhizome, Poria, Licorice Root, Moutan Bark, Gardenia Fruit, Mint Herb, Ginger*	To treat functional dyspepsia.
Shao-Yao-Gan-Cao-Tang	*Rx. Paeoniae Alba, Rx. Glycyrrhizae Preparata* *Paeonia lactiflora, Glycyrrhiza uralensis*	Antioxidative and antiaggregation effect
Liu-Wei-Di-Huang-Wan	*Rehmannia Root, Fructus Corni, Dioscorea Rhizome, Alisma Rhizome, Moutan cortex, Poria cocos.*	Liver and kidney yin dificiencies, soreness and weakness of the lower back and knees, dizziness, parched mouth and sore throat, heel pain. Enriches the yin and nourishes the kidneys.
Ge-Gen-Tang	*Puerariae radix (Pueraria lobata Ohwi), Ephedrae Herba (Ephedra sinica Stapf), Cinnamomi Ramulus (Cinnamomum cassia Blume), Paeoniae Radix (Paeonia lactiflora Pallas), Glycyrrhizae Radix preparata (Glycyrrhiza uralensis Fischer), Zingiberis Rhizoma (Zingiber officinale Roscoe), and Zizyphi Fructus (Ziziphus jujuba Mill. var. inermis Rehder)*	Induces sweating to release the exterior symptoms and dispel Wind-Cold. It is indicated for stiff neck, headache, muscle aches, alternating chills and fever, sneezing, cough, nasal congestion, runny nose.
Ji-Sheng-Shen-Qi-Wan	*Rehmannia Root, Fructus Corni, Dioscorea Rhizome, Alisma Rhizome, Moutan cortex, Poria cocos, Roasted Aconite, Cinnamon Bark, Achyranthes bidentata, Semen Plantaginis.*	Kidney yang deficiency, lower backache, urinary difficulties./Warms and tonifies kidney yang, regulates water and relieves edema.
Xiao-Qing-Long-Tang	*Herba Ephedrae, Rhizoma Zingiberis, Ramulus Cinnamomi, Radix et Rhizoma Asari, Rhizoma Pinellia, Fructus Schisandrae Chinensis, Radix et Rhizoma Glycyrrhizae, and Radix paeoniae alba.*	Used to treat bronchial asthma and allergic rhinitis.
Xue-Fu-Zhu-Yu-Tang	*Achyranthes bidentata Blume, Angelica sinensis Diels, radix, Citrus aurantium L., fructus, Bupleurum chinense DC., radix, Carthamus tinctorius L., flos, Glycyrrhiza uralensis Fisch., radix and rhizome, Ligusticum chuanxiong Hort., rhizome, Paeonia lactiflora Pall., radix, Prunus persica Stokes, semen, Platycodon grandifloras, A. DC., radix, Rehmannia glutinosa, Libosch., radix*	Invigorate Blood; Expel Blood Stasis; Move Qi; Stop Pain
Chuan-Xiong-Cha-Tiao-San	*Radix Chuanxiong, Herba Schizonepetae, Radix Saposhnikoviae, Rhizoma et Radix Notopterygii, Radix et Rhizoma Glycyrrhizae, Radix Angelicae Dahuricae, Herba Menthae, Radix et Rhizoma Asari*	Dispels wind and clears away heat. It is used for headache, migraine, or headache on the top, aversion to cold and fever, dizziness, nasal obstruction
Ping-Wei-San	*Atractylodes lancea rhizome, Magnolia officinalis bark, Citrus reticulata peel, Glycyrrhiza uralensis root, Zingiber officinale rhizome, Ziziphus jujuba fruit*	For gastroenteritis, intestinal obstruction, coronary artery disease and peptic ulcer
Ban-Xia-Xie-Xin-Tang	*Pinellia ternate, Makino, Panax ginseng, Zingiber officinale Roscoe, Coptis chinensis Franch., Scutellaria baicalensis Georgi, Ziziphus jujuba Mill.*	Treating metabolic diseases, such as nonalcohol fatty liver disease, diabetes mellitus, and obesity.

## Data Availability

All of the analytical data were retrieved from the National Health Insurance Research Database provided by the Bureau of National Health Insurance, managed by the Department of Health and Welfare, Taiwan. Because of restrictions imposed by the law of “Personal Information Protection Act”, the relevant data cannot be publicly obtained. Requisition for usage of datasets should be directed to the Bureau of National Health Insurance and the corresponding author.

## References

[1] Ong K.L., Stafford L.K., McLaughlin S.A., Boyko E.J., Vollset S.E., Smith A.E., Dalton B.E., Duprey J., Cruz J.A., Hagins H. (2023). Global, regional, and national burden of diabetes from 1990 to 2021, with projections of prevalence to 2050: A systematic analysis for the Global Burden of Disease Study 2021. Lancet.

[2] Sun H., Saeedi P., Karuranga S., Pinkepank M., Ogurtsova K., Duncan B.B., Stein C., Basit A., Chan J.C.N., Mbanya J.C. (2022). IDF Diabetes Atlas: Global, regional and country-level diabetes prevalence estimates for 2021 and projections for 2045. Diabetes Res. Clin. Pract..

[3] Parker E.D., Lin J., Mahoney T., Ume N., Yang G., Gabbay R.A., ElSayed N.A., Bannuru R.R. (2023). Economic Costs of Diabetes in the U.S. in 2022. Diabetes Care.

[4] Shafiepour M., Bamdad Z., Radman M. (2022). Prevalence of hearing loss among patients with type 2 diabetes. J. Med. Life.

[5] Horikawa C., Kodama S., Tanaka S., Fujihara K., Hirasawa R., Yachi Y., Shimano H., Yamada N., Saito K., Sone H. (2013). Diabetes and risk of hearing impairment in adults: A meta-analysis. J. Clin. Endocrinol. Metab..

[6] Sun J., Singh P., Österlund J., Orho-Melander M., Melander O., Engström G., Edsfeldt A. (2021). Hyperglycaemia-associated Caspase-3 predicts diabetes and coronary artery disease events. J. Intern. Med..

[7] Bhatti J.S., Sehrawat A., Mishra J., Sidhu I.S., Navik U., Khullar N., Kumar S., Bhatti G.K., Reddy P.H. (2022). Oxidative stress in the pathophysiology of type 2 diabetes and related complications: Current therapeutics strategies and future perspectives. Free Radic. Biol. Med..

[8] Liu T., Zhang L., Joo D., Sun S.-C. (2017). NF-κB signaling in inflammation. Signal Transduct. Target Ther..

[9] Samaha N.L., Almasri M.M., Johns J.D., Hoa M. (2021). Hearing restoration and the stria vascularis: Evidence for the role of the immune system in hearing restoration. Curr. Opin. Otolaryngol. Head Neck Surg..

[10] Watson N., Ding B., Zhu X., Frisina R.D. (2017). Chronic inflammation–inflammaging–in the ageing cochlea: A novel target for future presbycusis therapy. Ageing Res. Rev..

[11] Ahn J.H., Yang J.S., Jung J., Kang S., Jung S.J. (2024). Association between hearing loss and suicidal ideation: Discrepancy between pure tone audiometry and subjective hearing level. J. Affect. Disord..

[12] Li H., Hung A., Li M., Yang A.W.H. (2019). Fritillariae thunbergii bulbus: Traditional uses, phytochemistry, pharmacodynamics, pharmacokinetics and toxicity. Int. J. Mol. Sci..

[13] Li D., You H.J., Hu G.J., Yao R.Y., Xie A.M., Li X.Y. (2022). Mechanisms of the Ping-wei-san plus herbal decoction against Parkinson’s disease: Multiomics analyses. Front. Nutr..

[14] Xia Q.S., Gao Y., Wen-Bin W., Wu F., Dong H., Xu L.J., Fang K., Hu M.L., Yuan F., Lu F.E. (2022). Ban-xia-xie-xin-tang ameliorates hepatic steatosis by regulating Cidea and Cidec expression in HFD-fed mice. Phytomedicine.

[15] Tran N., Pham B., Le L. (2020). Bioactive compounds in anti-Diabetic plants: From herbal medicine to modern drug discovery. Biology.

[16] Yen C.-T., Livneh H., Huang H.-L., Lu M.-C., Chen W.-J., Tsai T.-Y. (2024). Decreased risk of osteoporosis incident in subjects receiving Chinese herbal medicine for Sjögren syndrome treatment: A retrospective cohort study with a nested case-control analysis. Pharmaceuticals.

[17] Shih C.C., Liao C.C., Su Y.C., Tsai C.C., Lin J.G. (2012). Gender differences in traditional Chinese medicine use among adults in Taiwan. PLoS ONE.

[18] Aljefree N.M., Almoraie N.M., Althaiban M.A., Hanbazaza M.A., Wazzan H.A., Shatwan I.M. (2023). Gender differences in knowledge, attitudes, and practices with respect to type 1 diabetes among Saudi public-school teachers. BMC Public Health.

[19] Williamson T.T., Ding B., Zhu X., Frisina R.D. (2019). Hormone replacement therapy attenuates hearing loss: Mechanisms involving estrogen and the IGF-1 pathway. Aging Cell.

[20] Khalin I., Alyautdin R., Kocherga G., Bakar M.A. (2015). Targeted delivery of brain-derived neurotrophic factor for the treatment of blindness and deafness. Int. J. Nanomed..

[21] Eldufani J., Blaise G. (2019). The role of acetylcholinesterase inhibitors such as neostigmine and rivastigmine on chronic pain and cognitive function in aging: A review of recent clinical applications. Alzheimers Dement.

[22] Kim J.H., Kim M., Hong S., Kwon B., Song M.W., Song K., Kim E.Y., Jung H.S., Sohn Y. (2021). Anti-inflammatory effects of Fritillaria thunbergii Miquel extracts in LPS-stimulated murine macrophage RAW 264.7 cells. Exp. Ther. Med..

[23] Hung Y.C., Pan T.L., Hu W.L. (2016). Roles of reactive oxygen Species in anticancer therapy with salvia miltiorrhiza bunge. Oxid. Med. Cell. Longev..

[24] Lin S.K., Tsai Y.T., Lai J.N., Wu C.T. (2015). Demographic and medication characteristics of traditional Chinese medicine users among dementia patients in Taiwan: A nationwide database study. J. Ethnopharmacol..

[25] Neubrech F., Sauerbier M., Moll W., Seegmüller J., Heider S., Harhaus L., Bickert B., Kneser U., Kremer T. (2018). Enhancing the outcome of traumatic sensory nerve lesions of the hand by additional use of a chitosan nerve tube in primary nerve repair: A randomized controlled bicentric trial. Plast. Reconstr. Surg..

[26] Ahn S.-I., Cho S., Choi N.-J. (2021). Effectiveness of chitosan as a dietary supplement in lowering cholesterol in murine models: A meta-analysis. Mar. Drugs.

[27] Tsai F.J., Ho T.J., Cheng C.F., Shiao Y.T., Chien W.K., Chen J.H., Liu X., Tsang H., Lin T.H., Liao C.C. (2017). Characteristics of Chinese herbal medicine usage in ischemic heart disease patients among type 2 diabetes and their protection against hydrogen peroxide-mediated apoptosis in H9C2 cardiomyoblasts. Oncotarget.

[28] Driver E.C., Kelley M.W. (2020). Development of the cochlea. Development.

[29] Shi F., Hu L., Jacques B.E., Mulvaney J.F., Dabdoub A., Edge A.S.B. (2014). β-Catenin is required for hair-cell differentiation in the cochlea. J. Neurosci..

[30] Bonetto S., Gruden G., Beccuti G., Ferro A., Saracco G.M., Pellicano R. (2021). Management of dyspepsia and gastroparesis in patients with diabetes. a clinical point of view in the year 2021. J. Clin. Med..

[31] Kim K., Ko S.J., Cho S.H., Kim J., Park J.W. (2023). Herbal medicine, Banxia-xiexin tang, for functional dyspepsia: A systematic review and meta-analysis. Front. Pharmacol..

[32] Chen H., Fu W., Chen H., You S., Liu X., Yang Y., Wei Y., Huang J., Rui W. (2019). Magnolol attenuates the inflammation and enhances phagocytosis through the activation of MAPK, NF-κB signal pathways in vitro and in vivo. Mol. Immunol..

[33] Zhang Z., Shen P., Xie W., Cao H., Liu J., Cao Y., Zhang N. (2019). Pingwei san ameliorates dextran sulfate sodium-induced chronic colitis in mice. J. Ethnopharmacol..

[34] Hu X., Sato J., Bajotto G., Khookhor O., Ohsawa I., Oshida Y., Sato Y. (2010). Goshajinkigan (Chinese herbal medicine niu-che-sen-qi-wan) improves insulin resistance in diabetic rats via the nitric oxide pathway. Nagoya J. Med. Sci..

[35] Xing Z., Xia Z., Peng W., Li J., Zhang C., Fu C., Tang T., Luo J., Zou Y., Fan R. (2016). Xuefu Zhuyu decoction, a traditional Chinese medicine, provides neuroprotection in a rat model of traumatic brain injury via an anti-inflammatory pathway. Sci. Rep..

[36] Che C.T., Wong M.S., Lam C.W. (2016). Natural products from Chinese medicines with potential benefits to bone health. Molecules.

[37] National Health Insurance Database, Taiwan. LHID 2000. https://dep.mohw.gov.tw/DOS/cp-5119-59201-113.html.

[38] Liu C.Y., Hung Y.T., Chuang Y.L., Chen Y.J., Weng W.S., Liu J.S., Liang K.Y. (2006). Incorporating development stratification of Taiwan townships into sampling design of large scale health interview survey (in Chinese). J. Health Manag..

[39] Deyo R.A., Cherkin D.C., Ciol M.A. (1992). Adapting a clinical comorbidity index for use with ICD-9-CM administrative databases. J. Clin. Epidemiol..

